# Prevention of Generalized Anxiety Disorder Using a Web Intervention, iChill: Randomized Controlled Trial

**DOI:** 10.2196/jmir.3507

**Published:** 2014-09-02

**Authors:** Helen Christensen, Philip Batterham, Andrew Mackinnon, Kathleen M Griffiths, Kanupriya Kalia Hehir, Justin Kenardy, John Gosling, Kylie Bennett

**Affiliations:** ^1^Black Dog InstituteUniversity of New South WalesSydneyAustralia; ^2^National Institute for Mental Health ResearchAustralian National UniversityCanberraAustralia; ^3^Orygen Youth Mental Health Research CentreUniversity of MelbourneMelbourneAustralia; ^4^Australian Primary Health Care Research InstituteAustralian National UniversityCanberraAustralia; ^5^Centre of National Research on Disability and Rehabilitation MedicineUniversity of Queensland Schools of Medicine and PsychologyBrisbaneAustralia

**Keywords:** anxiety disorders, prevention, early intervention, Internet, online systems, cognitive behavioral therapy

## Abstract

**Background:**

Generalized Anxiety Disorder (GAD) is a high prevalence, chronic disorder. Web-based interventions are acceptable, engaging, and can be delivered at scale. Few randomized controlled trials evaluate the effectiveness of prevention programs for anxiety, or the factors that improve effectiveness and engagement.

**Objective:**

The intent of the study was to evaluate the effectiveness of a Web-based program in preventing GAD symptoms in young adults, and to determine the role of telephone and email reminders.

**Methods:**

A 5-arm randomized controlled trial with 558 Internet users in the community, recruited via the Australian Electoral Roll, was conducted with 6- and 12-month follow-up. Five interventions were offered over a 10-week period. Group 1 (Active website) received a combined intervention of psycho-education, Internet-delivered Cognitive Behavioral Therapy (ICBT) for anxiety, physical activity promotion, and relaxation. Group 2 (Active website with telephone) received the identical Web program plus weekly telephone reminder calls. Group 3 (Active website with email) received the identical Web program plus weekly email reminders. Group 4 (Control) received a placebo website. Group 5 (Control with telephone) received the placebo website plus telephone calls. Main outcome measures were severity of anxiety symptoms as measured by the GAD 7-item scale (GAD-7) (at post-test, 6, and 12 months). Secondary measures were GAD caseness, measured by the Mini International Neuropsychiatric Interview (MINI) at 6 months, Centre for Epidemiologic Studies-Depression scale (CES-D), Anxiety Sensitivity Index (ASI), Penn State Worry Questionnaire (PSWQ), and Days out of Role.

**Results:**

GAD-7 symptoms reduced over post-test, 6-month, and 12-month follow-up. There were no significant differences between Group 4 (Control) and Groups 1 (Active website), 2 (Active website with telephone), 3 (Active website with email), or 5 (Control with telephone) at any follow-up. A total of 16 cases of GAD were identified at 6 months, comprising 6.7% (11/165) from the Active groups (1, 2, 3) and 4.5% (5/110) from the Control groups (4, 5), a difference that was not significant. CES-D, ASI, and PSWQ scores were significantly lower for the active website with email reminders at post-test, relative to the control website condition.

**Conclusions:**

Indicated prevention of GAD was not effective in reducing anxiety levels, measured by GAD-7. There were significant secondary effects for anxiety sensitivity, worry, and depression. Challenges for indicated prevention trials are discussed.

**Trial Registration:**

International Standard Randomized Controlled Trial Number (ISRCTN): 76298775; http://www.controlled-trials.com/ISRCTN76298775 (Archived by WebCite at http://www.webcitation.org/6S9aB5MAq).

## Introduction

Approximately 5% of the general population experiences General Anxiety Disorder (GAD) at least once in their lifetime [1], with population surveys indicating a lifetime prevalence rate of between 4.3-5.9% and a 12-month prevalence rate of between 1.2-1.9% [2,3]. The cost of GAD to the community is high as a result of its chronic course [4]. GAD frequently presents early in the lifespan and affects the individual throughout adulthood, with an estimated lag time to treatment of between 9 and 23 years [5]. If the prevalence of GAD is to be lowered, prevention, particularly focusing on the early adult and adolescence years when the illness emerges [6], will reduce the prevalence of mental disorder by up to 23% [7-9].

There is some evidence that GAD can be prevented. However, the conduct of research trials has not been optimal either because the researchers have been unable to exclude those with a diagnosis at the onset of the intervention or because the trials are too small or too short to investigate the number of incident cases following the intervention [10]. We reviewed the research literature, but found that only four trials excluded a diagnosis of GAD in adults at baseline. Two of the studies found preventative effects, but both trials had limitations [11,12]. Van’t Veer Tazelaar and colleagues [12] reported that depression and anxiety caseness could be halved in elderly people who were provided with a stepped care intervention of problem solving and cognitive behavioral therapy (CBT) bibliotherapy. However, the investigators did not report data separately for GAD, so the effect of the intervention on GAD compared to depression diagnosis could not be determined. Pitceathly and colleagues [13] reported a protective effect of a brief coping intervention on GAD in cancer patients. The effect was not detectable in the full sample, but was evident for those identified at the start of the trial with high risk of anxiety or depression. No preventative effects were found in the other two studies [14,15]. In the first, a stepped care intervention in elderly people living in residential care did not result in reduced incidence of combined anxiety or depression. In the second, carers of patients with Alzheimer’s disease did not show lower levels of anxiety or depression as a result of an intervention involving a Family Meetings intervention. In effect, no genuine prevention trials have been conducted with younger adults and none with adults without a cancer diagnosis.

A key challenge to delivering prevention interventions is the low level of engagement by those at risk; if symptoms are not disabling, motivation may be low and seeking help from doctors seen as inappropriate. Web-based interventions provide a potentially very useful delivery medium because they are accessible, acceptable, globally disseminable, and have been found to be effective in delivering CBT in clinical settings for both depression and anxiety [16-18]. Engagement may be enhanced by “push” factors (ie, factors that encourage involvement or engagement, such as reminders or coaching) [19]. However, inconsistent findings are reported [20-23]. Because prevention programs are delivered to large numbers at a population level, the costs associated with different push factors are critical to the feasibility of prevention efforts. Hence, there is a need to know the extent to which email reminders and telephone communication with the research team will improve adherence and effectiveness. The present study aimed to evaluate the effectiveness of a Web-based multimedia CBT intervention in individuals aged 18 to 30 years with symptoms of anxiety, who did not meet diagnostic criteria at baseline. The intervention was a website that provided psychoeducation, CBT, physical activity promotion, and relaxation training, with the majority of the sessions focusing on CBT. Each component of the intervention was found to be effective in GAD treatment [24-27]. Our rationale to include all four components was based on the view that combining a range of evidence-based interventions provides potential for maximal impact, as well as the opportunity for participants’ preferences (for example, see [28]). We know very little about engagement in prevention (compared to treatment) programs, and offering a range of interventions, all of which have evidence-based support from treatment settings, would potentially optimize effectiveness and uptake. We focused on young adults, as GAD develops during adolescence and early adulthood and any improvement would be likely to provide benefits over years. The intervention’s “e-couch” website is open following registration and the CBT “worry” program can be experienced.

Five interventions were offered over a 10-week period. Group 1 (Active website) received the combined intervention as described above. Group 2 (Active website with telephone) received the same Web program plus weekly telephone reminder calls. Group 3 (Active website with email) received the Web program plus automated weekly email reminders. Group 4 (Control) received a placebo website, matched in length to the active website. Group 5 (Control with telephone) received the placebo website plus telephone reminder calls. This design allowed us to compare the effectiveness of the active interventions to the control condition (1, 2, 3 vs 4) and also to determine the independent effect of phone contact in the control conditions (5 vs 4). Email reminders are less expensive than person-made telephone reminders, an important consideration for a prevention trial. To our knowledge, no similar prevention trials have been conducted with each of these inclusions: the use of an online intervention for GAD, targeting of young adults in the community, and excluding existing GAD diagnosis.

## Methods

### Study Design

A randomized controlled trial with 5 arms, called the “iChill” trial, with post-test, 6- and 12-month follow-up, was conducted. The study was approved by the ANU Human Ethics Committee (Protocol 2008/548).

### Setting, Participants, and Eligibility Criteria

The study protocol [29] describes trial details. A screening questionnaire was emailed to 120,000 randomly chosen Australians aged 18-30 years registered on the Australian Electoral Roll. Individuals meeting inclusion criteria were invited to a Web portal where they provided consent and undertook screening and baseline surveys. They were then interviewed via telephone to determine current GAD diagnosis using the MINI International Neuropsychiatric Interview (MINI) [30] and randomized to the trial. Inclusion criteria were willingness to consent, an active email and phone number, English language proficiency, Internet access, and a score above 5 on the GAD-7 [31]. In order to specify the target population for whom the intervention might be effective, participants were excluded if they were currently undergoing CBT or seeing a psychologist or a psychiatrist, had a current or previous diagnosis of bipolar disorder, schizophrenia, or psychosis, were at risk of self-harm or suicide based on the MINI depression module, or had a current diagnosis of panic disorder, social phobia, or post-traumatic stress disorder (PTSD) on the MINI. Participants were not excluded if taking antidepressants or benzodiazepines. A total of 40 (7.2%, 40/558) were taking antidepressants and 8 (1.4%, 8/558) were taking benzodiazepines at baseline; 510 were not taking either antidepressants or benzodiazepines.

### Randomization

The algorithm for randomization consisted of a stratified block design with eight strata (2 x 2 x 2) corresponding to gender, past GAD diagnosis, and severity of GAD symptoms with a block size of 10. “Past GAD diagnosis” was obtained from the MINI—a proportion of participants (24.2%, 135/558) met lifetime criteria for GAD on the MINI but did not meet MINI criteria for current GAD. To minimize imbalance between such participants, stratification was conducted on the basis of meeting lifetime GAD criteria (along with gender and current GAD symptoms). Allocation was administered via software architecture, and participants were informed of condition allocation after baseline interview.

### Interventions

The Active website intervention was a 10-week structured version of the Anxiety and Worry modules of the “e-couch” program (ecouch.anu.edu.au), which consisted of an integrated program of psychoeducation (weeks 1-2), CBT (weeks 3-7), relaxation (weeks 8-9), and physical activity promotion (week 10). The psychoeducation section (Modules 1 and 2) provides information on worry, stress, fear, and anxiety; a description of anxious thinking; differentiation of GAD from other anxiety disorders; risk factors for GAD; comorbidity; and consequences of anxiety and available treatments. This section is based on interventions for mental health literacy that have succeeded in reducing symptoms of depression and anxiety, and improving mental health attitudes [32]. The CBT toolkits (Modules 3-7) addressed typical anxious thoughts and included sections on dealing with the purpose and meaning of worry, the act of worrying, and the content of worry. The information is derived from materials that have been found to reduce anxious cognitions in at-risk people [33,34]. Progressive muscle relaxation (PMR) (Module 8) instructs participants on how to progressively tense and relax different muscle groups to induce relaxation and help to identify tension early. PMR has been trialed in a previous website program for depression in adults [35] and adolescents [36]. The mindfulness meditation module (Module 9) helps participants become aware of their breathing and body, acknowledging thoughts and external distractions but remaining focused on the present. The final module, physical activity (Module 10), tailors advice about physical activity based on the stages of change theory [37]. The control website was an adapted version of the HealthWatch control condition developed for the Australian National University WellBeing study [38]. This website provided information about general health (nutrition, heart health, etc), and invited responses to questions about anxiety. Scripted telephone reminder calls in the Active plus telephone condition were made on a weekly basis to check on participants’ progress and to remind them to complete the module and/ or to keep completing the program. The phone reminders were intended to serve purely as reminders, had no therapeutic input, and were made by casual phone interviewers. Telephone calls were scripted and were based on the email scripts. Any technical issues were referred to the Trial Manager. Phone calls generally lasted between 30 seconds to 2 minutes. Phone calls were made regardless of whether the participant completed the program or not. Participants in the Active plus email and Control plus email conditions were sent a weekly reminder email. These were similar in content to the phone calls. There was no therapeutic input.

### Outcome Measures

The primary outcome was the Generalized Anxiety Disorder 7-item scale (GAD-7) [31]. Secondary outcomes were GAD caseness based on MINI; worry, measured by the Penn State Worry Questionnaire (PSWQ) [39]; anxiety sensitivity, as measured by the Anxiety Sensitivity Index (ASI) [40]; depression symptoms, measured by the Centre for Epidemiologic Studies-Depression Scale (CES-D) [41]; and disability measured by Days out of Role from the US National Comorbidity Study [42]. GAD caseness was measured at 6 months. GAD caseness at 36 months will be determined by proxy using GAD-7 cut-off scores. Other measures not analyzed in this paper focused on comorbidities, such as harmful/hazardous alcohol use as measured by the Alcohol Use Disorders Identification Test (AUDIT) [43], or duplicate measures of depression caseness estimated by the Patient Health Questionnaire 9-item (PHQ-9) [44], and other behaviors such as help seeking and perceived need for treatment. Outcomes were assessed at baseline, 6, and 12 months, with the exception of MINI caseness, which was assessed at 6 months.

### Sample Size

We aimed, conservatively, to find an effect of 0.3 between each Active website group and the Control, based on effect sizes of 0.6 found for previous treatment and indicated prevention trials (0.6) [33,34]. This assumes a pre-post correlation of .7 between scores. With 600 participants, we would have 80% power to detect effects, allowing for 15% attrition.

### Statistical Analysis

Primary analyses were undertaken on an intent-to-treat basis (ITT). Mixed model repeated measures (MMRM) [45] were used to include all available data, including that from participants who subsequently withdrew from the trial. This approaches yields unbiased estimates of intervention effects under the assumption that data are missing at random (MAR). Unlike conventional approaches to analysis, MAR allows observations to be missing conditional on observed variables appearing in the analytic model [46]. Non-linear mixed models were used to analyze caseness.

## Results

**Figure 1:**
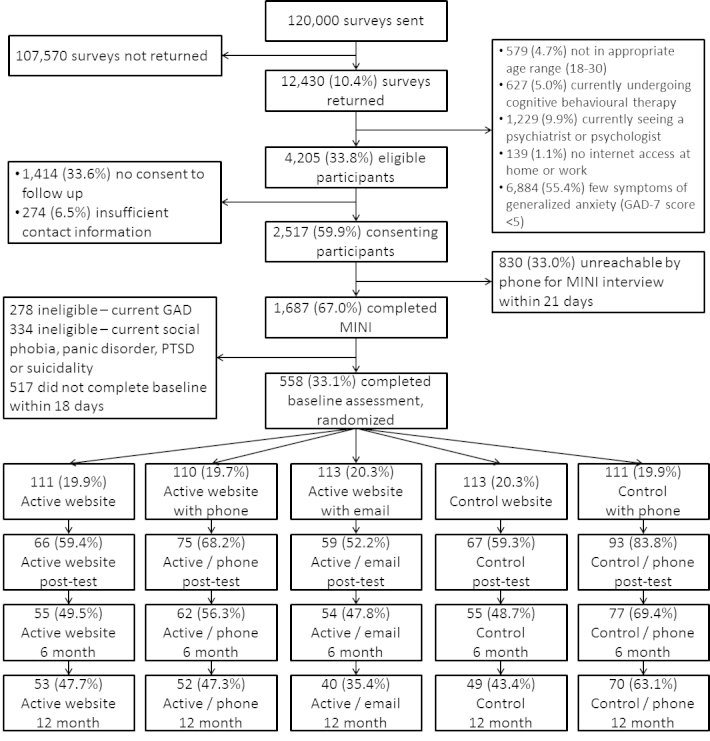
CONSORT diagram showing flow of participants through the study.

A total of 558 people were randomized to a trial condition of whom 360 (64.5%) completed post-test, 303 (54.3%) completed 6-month follow-up, and 264 (47.3%) completed the 12-month follow-up. Figure 1 shows the flow of participants. Sample characteristics are presented in Table 1. Although mean GAD-7 scores were above the cut-point of 5 at screening for all participants (mean 8.3, SD 3.3), anxiety symptoms had decreased by the time the baseline was completed (mean 6.7, SD 3.8).

There were no differences on any of the baseline measures with the exception of “preference for active condition” and “employment status”. Across all conditions, the most preferred program was the Active website with email reminders (37.8%, 211/558), followed by the Control (31.4%, 175/558), with few participants stating a preference for telephone reminders (5.6%, 31/558 and 11.6%, 65/558, for Active and Control conditions, respectively). Lower preference for the Active website was found among those in the Control and Active with email conditions. Higher rates of full-time work were found among those receiving the Active website with telephone reminders and lower rates among those receiving the Control website with telephone reminders.

Adherence to the intervention differed significantly according to condition. Participants in the reminder conditions completed the majority of the 10 modules (Active/email: 5.5 modules, Active/telephone: 7.3, Control/telephone: 8.3) while those who did not receive reminders completed a little over one-third (3.7 modules for both active and control; *F*_4, 477.5_=38.1, *P*<.001).

**Figure 2:**
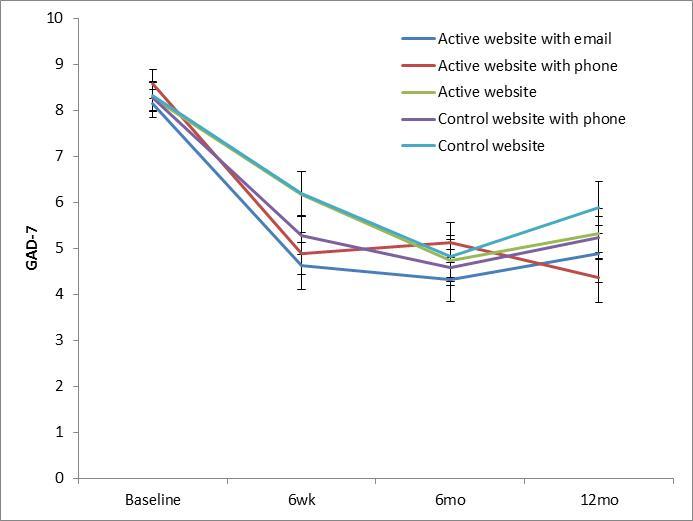
Outcome data on GAD-7 at post-test, 6-month and 12-month follow-ups (error bars represent standard error).

GAD-7 symptoms reduced at post-test and 6-month follow-up, but returned to baseline levels by 12 months. There were no significant differences between Group 4 (Control) and Groups 1 (Active website), 2 (Active website with telephone), 3 (Active website with email), or 5 (Control with telephone) at any follow-up. Outcomes were unchanged after adjusting for employment status and preferences for condition. Likewise, accounting for adherence by adjusting for module completion (ie, testing the efficacy of the intervention) did not change these outcomes. Figure 2 shows estimated marginal means of GAD-7 scores from the mixed model shown in Table 2.

Data on secondary outcomes analyses are displayed for continuous variables in Table 3. Based on the MINI assessment at 6 months, 16 cases of GAD were identified, comprising 6.7% from the Active groups (1, 2, and 3) and 4.5% from the Control groups (4, 5). There was no significant difference in the number of cases across these collapsed groups.

The mixed effect model repeated measure (MMRM) analyses for secondary outcomes were as follows. As for the primary outcome, there were no significant overall interactions between condition and time for CES-D, PSWQ, or Days out of Role. However, there was a significant interaction between condition and time for the ASI. Furthermore, there were significant effects of specific conditions at specific time points. CES-D, ASI, and PSWQ scores were significantly lower for the active website with email reminders at post-test, relative to the control website condition (*t*_389.2_= −2.5, *P*=.015; *t*_368.7_=−3.4, *P*<.001; *t*_371.9_=−2.4, *P*=.017 respectively). The decrease in ASI scores for the active/email condition remained significant at 6 months (*t*_343.1_=−2.3, *P*=.021). In addition, Days out of Role due to anxiety was significantly decreased at 12 months (but not at post-test or 6 months) for the active/email condition (*t*_398.3_=−2.4, *P*=.016). There was also reduced worry in the active website with phone reminders at post-test and 6 months, relative to the control condition (*t*_368.6_=−2.0, *P*=.047; *t*_340.2_= −2.1, *P*=.035 respectively).

**Table 1:** Characteristics of the sample at baseline by trial arm.

		Active website (n=111)	Active website with email (n=113)	Active website with phone (n=110)	Control website (n=111)	Control website with phone (n=113)	*F* ^a^ or *χ2*	*P*
		mean (SD) or n (%)						
**Characteristic**								
	Age	25.7 (3.2)	25.4 (3.3)	25.5 (3.1)	26.0 (3.0)	25.6 (3.5)	0.623	.646
	GAD-7^d^ score	6.8 (3.9)	6.2 (3.9)	6.8 (3.6)	6.9 (3.8)	6.6 (3.7)	0.604	.660
	CES-D^e^ score	16.7 (10.1)	17.7 (10.9)	16.7 (9.8)	18.8 (10.6)	17.3 (8.8)	0.802	.524
	Anxiety sensitivity	19.3 (11.0)	19.9 (11.5)	17.5 (10.6)	18.7 (9.9)	18.8 (9.9)	0.753	.556
	PSWQ^f^	40.5 (12.2)	37.9 (12.5)	39.5 (11.6)	40.3 (12.0)	39.2 (10.8)	0.835	.503
	AUDIT^g^ score	7.2 (5.1)	7.2 (5.7)	6.7 (4.8)	6.9 (4.7)	7.0 (5.3)	0.189	.944
	DOR^h^ due to anxiety	0.6 (1.5)	0.9 (2.3)	0.6 (1.6)	0.6 (1.4)	0.6 (1.2)	0.859	.488
	Self-rated health^i^	2.4 (0.9)	2.5 (0.8)	2.4 (0.8)	2.5 (0.9)	2.5 (0.7)	0.879	.476
	Childhood adversity^j^	1.5 (1.4)	1.9 (1.5)	1.8 (1.6)	1.7 (1.5)	1.7 (1.5)	0.919	.452
	Traumatic events^k^	1.4 (1.5)	1.6 (1.6)	1.5 (1.5)	1.4 (1.5)	1.6 (1.6)	0.498	.737
	Positive beliefs Internet therapy^l^	5.6 (1.2)	5.4 (1.3)	5.4 (1.3)	5.5 (1.3)	5.8 (1.3)	2.064	.084
	Female gender	92 (82.9%)	90 (79.6%)	88 (80.0%)	89 (80.2%)	91 (80.5%)	0.474^b^	.976
	Completed university degree	37 (33.3%)	32 (28.6%)	28 (25.9%)	36 (32.4%)	30 (26.8%)	2.352^b^	.671
	Prefer active condition^m^	67 (60.4%)	51 (45.1%)	70 (63.6%)	60 (54.1%)	70 (61.9%)	10.501^b^	.033^c^
**Employment status**							16.297^b^	.038^c^
	Full-time employment	69 (62.2%)	68 (61.3%)	81 (75.7%)	65 (59.1%)	58 (51.8%)		
	Part-time employment	30 (27.0%)	27 (24.3%)	16 (15.0%)	34 (30.9%)	36 (32.1%)		
	Not in labor force	12 (10.8%)	16 (14.4%)	10 (9.3%)	11 (10.0%)	18 (16.1%)		

^a^ *F* tests are from one-way analysis of variance (ANOVA) for continuous variables

^b^ *χ2* tests for categorical variables

^c^ *P*<.05

^d^GAD: Generalized Anxiety Disorder

^e^CES-D: Center for Epidemiologic Studies Depression Scale

^f^PSWQ: Penn State Worry Questionnaire

^g^AUDIT: Alcohol Use Disorders Identification Test

^h^DOR: Days out of Role

^i^Self-rated health assessed on a 5-point scale from 1 (excellent) to 5 (poor)

^j^Childhood adversity based on aggregate of 6 items assessing paternal/maternal mental health problems and substance use problems, high familial conflict, and parental separation/divorce

^k^Traumatic life events based on count from list of 14 traumatic events

^l^Positive beliefs in Internet therapy based on 2 items regarding confidence in learning skills about anxiety and better understanding anxiety using the Internet

^m^Preference for active condition based on a single item asking which intervention would be preferred

**Table 2:** Repeated measures mixed model of GAD-7 scores at post-test, 6, and 12 months.

Parameter		Estimate	Standard error	df	*t* / *F*	*P*
Intercept		6.946	0.359	553.0	19.3	<.001
**Condition**					1.0	.410
	Active website	−0.117	0.508	553.0	−0.2	.818
	Active website with email reminders	−0.716	0.506	553.0	−1.4	.158
	Active website with phone reminders	−0.173	0.510	553.0	−0.3	.734
	Control website with phone reminders	−0.335	0.506	553.0	−0.7	.508
	Control website	0.000	0.000			
**Time**					29.5	<.001
	Baseline	0.000	0.000			
	Post-test	−1.066	0.494	389.2	−2.2	.032
	6-month follow-up	−2.337	0.512	372.8	−4.6	<.001
	12-month follow-up	−1.178	0.612	315.8	−1.9	.055
**Condition × time interaction**					0.8	.622
	Active vs control at baseline	0.000	0.000			
	Active vs control at post-test	0.331	0.698	390.6	0.5	.636
	Active vs control at 6 months	0.179	0.723	374.2	0.2	.805
	Active vs control at 12 months	−0.274	0.853	313.9	−0.3	.748
	Active email vs control at baseline	0.000	0.000			
	Active email vs control at post-test	−0.517	0.718	393.9	−0.7	.472
	Active email vs control at 6 months	0.370	0.730	372.3	0.5	.612
	Active email vs control at 12 months	−0.157	0.896	319.4	−0.2	.861
	Active phone vs control at baseline	0.000	0.000			
	Active phone vs control at post-test	−0.689	0.682	384.5	−1.0	.314
	Active phone vs control at 6 months	0.736	0.709	369.4	1.0	.300
	Active phone vs control at 12 months	−1.082	0.851	315.4	−1.3	.205
	Control phone vs control at baseline	0.000	0.000			
	Control phone vs control at post-test	−0.137	0.655	379.2	−0.2	.835
	Control phone vs control at 6 months	0.394	0.683	364.4	0.6	.565
	Control phone vs control at 12 months	−0.150	0.808	310.2	−0.2	.853

**Table 3:** Primary and secondary outcome data.

Outcome		Active	Active / phone	Active / email	Control	Control / phone			
		mean (SD)/n	mean (SD)/n	mean (SD)/n	mean (SD)/n	mean (SD)/n	*F* ^a^	df	*P*
**Sample size**									
	Baseline	111	110	113	111	113			
	Post-test	66	75	58	66	93			
	6-month	55	62	54	55	77			
	12-month	53	52	40	48	70			
**GAD-7** ^c^							0.8	12, 323.4	.622
	Baseline	6.8 (3.9)	6.8 (3.6)	6.2 (3.9)	7.0 (3.8)	6.6 (3.7)			
	Post-test	6.1 (4.7)	4.7 (3.6)	4.6 (2.9)	6.1 (4.1)	5.3 (4.2)			
	6-month	4.3 (3.0)	5.0 (4.0)	4.3 (3.7)	4.6 (3.6)	4.7 (3.1)			
	12-month	5.1 (4.6)	4.0 (3.4)	5.1 (4.1)	5.9 (4.5)	5.1 (3.6)			
**PSWQ** ^d^							1.0	12, 345.4	.419
	Baseline	40.5 (12.2)	39.5 (11.6)	37.9 (12.5)	40.3 (12.0)	39.2 (10.8)			
	Post-test	39.0 (13.2)	37.4 (10.6)	33.8 (11.5)	41.0 (12.3)	38.4 (12.8)			
	6-month	33.9 (13.2)	38.2 (11.3)	35.9 (11.5)	38.9 (13.4)	37.9 (13.5)			
	12-month	34.1 (14.0)	33.2 (11.2)	34.4 (13.1)	38.3 (13.9)	37.2 (12.1)			
**ASI** ^e^							1.7	12, 345.9	.057
	Baseline	19.3 (11.0)	17.5 (10.6)	19.9 (11.5)	18.7 (9.9)	18.8 (9.9)			
	Post-test	18.9 (11.8)	14.6 (10.6)	17.1 (11.1)	18.5 (12.2)	16.7 (11.0)			
	6-month	16.0 (12.3)	15.0 (11.5)	15.4 (9.5)	17.4 (10.2)	15.1 (9.8)			
	12-month	19.5 (12.4)	14.7 (10.0)	19.4 (11.6)	20.3 (11.1)	21.1 (10.6)			
**CES-D** ^f^							1.9	12, 328.4	.036^b^
	Baseline	16.7 (10.1)	16.7 (9.8)	17.7 (10.9)	18.8 (10.6)	17.3 (8.8)			
	Post-test	14.0 (10.8)	12.4 (8.6)	10.9 (8.4)	17.5 (11.3)	13.8 (9.6)			
	6-month	10.7 (7.7)	12.6 (10.5)	11.8 (8.9)	14.4 (11.3)	13.4 (8.1)			
	12-month	12.0 (9.2)	9.7 (5.9)	12.3 (11.3)	15.3 (9.3)	12.6 (8.7)			
**DOR** ^g^							1.1	12, 369.9	.324
	Baseline	0.6 (1.5)	0.6 (1.6)	0.9 (2.3)	0.6 (1.4)	0.6 (1.2)			
	Post-test	0.5 (1.3)	0.2 (0.5)	0.4 (0.9)	0.5 (1.4)	0.2 (0.8)			
	6-month	0.3 (1.4)	0.4 (1.1)	0.1 (0.6)	0.5 (1.8)	0.5 (2.5)			
	12-month	0.3 (0.8)	0.2 (0.6)	0.1 (0.5)	0.7 (1.8)	0.4 (1.9)			

^a^statistics are omnibus *F* tests from mixed models repeated measures for each outcome, based on time × condition interaction terms

^b^ *P*<.05

^c^GAD: Generalized Anxiety Disorder

^d^PSWQ: Penn State Worry Questionnaire

^e^ASI: Anxiety Sensitivity Index

^f^CES-D: Center for Epidemiologic Studies Depression scale

^g^DOR: days out of role due to anxiety

## Discussion

### Principal Findings

We found no evidence that a range of brief active interventions were associated with improved anxiety outcomes at post-test or at 6 or 12 months as measured by GAD-7 and by caseness at 6 months as measured by the MINI. We found that there were effects on secondary outcomes, most strongly found for anxiety sensitivity, where there was a significant interaction between condition and time for the ASI. In addition, ASI scores were significantly lower for the active website with email reminders at post-test, and at 6 months. There were also small effects at various follow-up intervals for the other measures mostly associated with the active website with email reminders. For instance, CES-D and PSWQ scores were significantly lower for the active website with email reminders at post-test, relative to the control website condition. In addition, Days out of Role due to anxiety was significantly decreased at 12 months for the active/email condition.

At best, these findings suggest that an active website with email may have small effects on a number of secondary outcomes. It is also possible that the GAD-7 may be a poorer measure of anxiety change than the ASI, and that genuine prevention effects operate, but were not discoverable because of our choice of outcome measure. The GAD-7 may be sensitive to a range of anxiety disorders [47] and may not have been the most robust outcome measure.

### Limitations

In addition, a number of limitations to the present study need to be considered. There was differential dropout in a number of conditions relative to the control. Nevertheless, the completer analyses (not reported in the paper but undertaken) produced comparable effects to the main ITT analysis. The condition with the strongest secondary outcome effects was also the condition that was associated with the highest preference rating, suggesting that preference for it might have influenced the findings. In addition, the intervention itself may not have been optimal. We argued that combining a range of evidence-based interventions would provide maximal impact as well as provide opportunity for participants to make choices within the program content. It might be suggested that this diluted the effects of the individual components. However, we disagree, given that (1) the combined intervention is highly efficacious (effect sizes greater than 1.0) for patients with a diagnosis of GAD [48], (2) the multimedia intervention in the current paper was associated with effects on a range of secondary measures, and (3) evidence from earlier online trials indicates that much shorter interventions (eg, over 5 sessions rather than 8) are associated with positive outcomes, and that very short interventions can be effective [49]. Consequently, we doubt that the intervention website itself was the reason for the lack of effect on the primary outcome measure. Another criticism is that choosing a multi-modal treatment results in the findings not being clear-cut, as any component might be the effective one. However, we would argue that prevention trials, in direct comparison to treatment trials, are in their infancy. Trials that demonstrate the effectiveness of multi-component interventions represent the first stage of a prevention research program, where, once an effect can be demonstrated, further research would normally then investigate the effect of the subcomponents.

The number of individuals in the current prevention study developing a diagnosis over the 12-month period following the intervention was unexpectedly low. However, elsewhere, comparable rates of 8.6% for the intervention and 4.44% in usual care groups have been reported [14]. These low rates may be due to regression to the mean and to the low threshold of anxiety for recruitment to the trial. The 6-month interval to determine caseness is relatively short with respect to prevention trials in the sense that a longer interval permits more opportunity to develop a disorder and thus to judge the effectiveness of the interventions. The choice of the 6-month interval for the MINI was made in order to maximize follow-up numbers (ie, to determine caseness) as dropouts were expected to increase over the 12-month follow-up period. We reasoned that we could determine proxy measures of caseness at 12 months via the GAD-7. A planned 3-year follow-up using GAD-7 as the primary outcome has commenced and we will report anxiety and depression outcomes at this time. Attrition was higher than expected at about 35% at post-test, although similar rates of dropout have been reported for face-to-face CBT [50].

Consistent with previous eHealth trials, data completion was higher in the control condition and was lowest for the active website condition with automated emails—a finding consistent with earlier trials [35,51]). The lower attrition in the control groups has been attributed to participant burden. Whether online or face-to-face, psychological interventions can be hard work, even threatening, and are often associated with dropout [52]. We also had substantial loss to recruitment between an expression of interest to the trial, and enrollment following invitation to undertake baseline measures. The reasons for failure to take up enrollment are not clear, but the trial was configured to allow enrollment within a week of consent. Delays in telephone and email contact often stretched the recruitment process, although there were strict time limits on each of the processes involved. The multi-modal nature of recruitment made identification of the reasons for non-response very complex, as the screener was conducted by post, the MINI assessment by telephone call, and the baseline invitation by email to an online survey.

A related issue is the role of contact in promoting adherence. In the present trial, adherence to the website was increased by contact via email or telephone. However, increased contact does not always result in improved adherence. A study we undertook with crisis call centers showed that adherence was much lower in participants who were provided with a website and telephone support compared to individuals without such support [53]. More research is required to examine for whom and under what circumstances telephone contact can increase adherence, and the factors that lead to increased dropout. We have reviewed factors that predict adherence for online programs [54]. We also acknowledge that the effects of the intervention in an adolescent rather than a young adult sample might have been more evident, since GAD or worry might emerge in this period. Levels of attrition need to be considered, since these were high. In addition, the sample excluded concurrent other diagnoses such as social phobia and PTSD, reducing generalizability. The trial raises the important question of how best to keep symptomatic people engaged in interventions. One possibility is to change orientation toward healthy living and offer prevention for GAD by stealth. An alternative approach is to constrain participants from dropping out through structure (eg, curriculum activities in schools or induction programs in workforces). These possibilities are currently being pursued in other research projects.

### Conclusions

Despite a number of limitations, the present trial represents a methodologically rigorous, well-executed prevention trial, which for the first time examines the effectiveness of the prevention of GAD in symptomatic 18-30 year olds in the community using online technologies. Diagnosis was established using a telephone interview at baseline and 6 months, and GAD diagnosis was an exclusion factor at commencement, ensuring that it was a genuine prevention trial. A post-hoc power analysis found that we had approximately 95% power to find a between-groups effect of *d*=0.3 between the Active website alone and Control website alone, indicating that the trial was sufficiently powered. Preference for trial condition was measured and assessed for its effect, and the study aimed to determine push and pull factors that might influence uptake and efficaciousness. In this trial, we were not able to demonstrate the preventative effects of the website on anxiety symptoms as measured by the GAD-7. There were indications that prevention was operating in one of the five conditions (email plus active website) on a number of the secondary measures. The 3-year follow-up will provide a stronger test of whether secondary outcomes such as anxiety sensitivity are modifiable in response to a website with email reminders and to determine whether anxiety symptoms and caseness are averted with a longer lapse of time.

## Multimedia Appendix 1

CONSORT-EHEALTH checklist V1.6.2 [55].

## Abbreviations

ASI: Anxiety Sensitivity Index
AUDIT: Alcohol Use Disorders Identification Test
CBT: Cognitive Behavioral Therapy
CES-D: Centre for Epidemiologic Studies-Depression
GAD: generalized anxiety disorder
ITT: intent-to-treat
MAR: missing at random
MINI: Mini International Neuropsychiatric Interview
PHQ-9: Patient Health Questionnaire 9-item
PMR: progressive muscle relaxation
PSWQ: Penn State Worry Questionnaire
