# Supplementary material for: Prevention of Generalized Anxiety Disorder Using a Web Intervention, iChill: Randomized Controlled Trial
Source: J Med Internet Res. 2014 Sep 2;16(9):e199. doi: 10.2196/jmir.3507 (PMC4211086; doi:10.2196/jmir.3507)
Supplement: Supplementary file 1 [file jmir_v16i9e199_app1.pdf]

CONSORT-EHEALTH Checklist V1.6.2 Report

(based on CONSORT-EHEALTH V1.6), available at [http://tinyurl.com/consort-ehealth-v1-6].

3507

Date completed

5/1/2014 2:04:06

by

Helen Christensen

The Prevention of Generalised Anxiety Disorder in those at risk using a Web Intervention: I-Chill, a Randomised Controlled Trial

TITLE

1a-i) Identify the mode of delivery in the title

"using a Web Intervention"

1a-ii) Non-web-based components or important co-interventions in title

None mentioned in the title.

1a-iii) Primary condition or target group in the title

From the title: "Generalised Anxiety Disorder in those at Risk".
We are targeting those at risk of developing GAD.

ABSTRACT

1b-i) Key features/functionalities/components of the intervention and comparator in the METHODS section of the ABSTRACT

"INTERVENTIONS: Five interventions were offered over a 10-week period. Group 1 (Active website) received a combined intervention of psycho-education, internet delivered Cognitive Behavioural Therapy for anxiety (iCBT), physical activity promotion, and relaxation. Group 2 (Active website with telephone) received the identical web program plus weekly telephone reminder calls. Group 3 (Active website with email) received the identical web program, plus weekly email reminders. Group 4 (Control) received a placebo website. Group 5 (Control with telephone) received the placebo website plus telephone calls."

1b-ii) Level of human involvement in the METHODS section of the ABSTRACT

No face-to-face human contact, as the internet program was self-accessed by participants.

However, Group 2 and Group 5 both received a web program "plus weekly telephone reminder calls"

1b-iii) Open vs. closed, web-based (self-assessment) vs. face-to-face assessments in the METHODS section of the ABSTRACT

Participants were "558 Internet users in the community, recruited via the Australian Electoral Roll was conducted with 6- and 12-month follow-up."

Purely web-based trial, plus telephone reminders for certain groups. No face-to-face components/

Outcomes were self-assessed through questionnaires.

This trial was unblinded as participants were informed of condition allocation after baseline interview.

1b-iv) RESULTS section in abstract must contain use data

"RESULTS: GAD-7 symptoms reduced over post-test, 6-month and 12-month follow-up. There were no significant differences between Group 4 (Control) and Groups 1 (Active website), 3 (Active website with email) or 5 (Control with telephone) at any follow-up. However, Group 2 (Active website with telephone) was associated with fewer symptoms at post-test and at 12 months. 16 cases of Generalised Anxiety Disorder were identified at six months, comprising 6.7% from the Active groups (1, 2, 3) and 4.5% from the Control groups (4, 5), a difference that was not significant. Both the Active website with telephone and Active website with email significantly differed from Control at various follow-up periods on ASI and Days out of Role."

1b-v) CONCLUSIONS/DISCUSSION in abstract for negative trials

"CONCLUSIONS: An intervention of an active website plus telephone reminders reduces anxiety symptoms at 12 months compared to an attention placebo. The effect is small."

INTRODUCTION

2a-i) Problem and the type of system/solution

"There is some evidence that GAD can be prevented. However, very few programs have excluded those with a diagnosis at the onset of the intervention, and few have investigated the reduction in the number of incident cases[...] A key challenge to delivering prevention interventions is the low level of engagement by those at risk; if symptoms are not disabling, motivation may be low, and seeking help from doctors seen as inappropriate."

2a-ii) Scientific background, rationale: What is known about the (type of) system

"Web-based interventions provide a potentially very useful delivery medium because they are accessible, acceptable, globally disseminable, and have been found to be effective in delivering CBT in clinical settings for both depression and anxiety. Engagement may be enhanced by “push” factors (i.e., factors that encourage involvement or engagement, such as reminders or coaching). However, inconsistent findings are reported. Because prevention programs are delivered to large numbers at a population level, the costs associated with different push factors are critical to the feasibility of prevention efforts. Hence, there is a need to know the extent to which email reminders and telephone communication with the research team will improve adherence and effectiveness."

METHODS

3a) CONSORT: Description of trial design (such as parallel, factorial) including allocation ratio

"The present study aimed to evaluate the effectiveness of an Internet-based multimedia CBT intervention in individuals aged 18 to 30 years with symptoms of anxiety, who did not meet diagnostic criteria at baseline."

3b) CONSORT: Important changes to methods after trial commencement (such as eligibility criteria), with reasons

None.

3b-i) Bug fixes, Downtimes, Content Changes

4a) CONSORT: Eligibility criteria for participants

Eligibility criteria:

"Inclusion criteria were willingness to consent, an active email and phone number, English language proficiency, internet access and a score above 5 on the GAD-730. Participants were excluded if they were currently undergoing CBT or seeing a psychologist or a psychiatrist, had a current or previous diagnosis of Bipolar Disorder, Schizophrenia, or Psychosis, were at risk of self-harm or suicide based on the MINI Depression module, had a current diagnosis of Panic Disorder, Social Phobia, or PTSD on the MINI."

4a-i) Computer / Internet literacy

Eligibility criteria: an active email account.

4a-ii) Open vs. closed, web-based vs. face-to-face assessments:

From the Methods: "A screening questionnaire was emailed to 120,000 randomly chosen Australians aged 18-30 years registered on the Australian Electoral Roll."

The intervention conditions for this trial were purely web-based, with certain conditions also including telephone calls by the trial administrators, but no face-to-face contact.

4a-iii) Information giving during recruitment

4b) CONSORT: Settings and locations where the data were collected

"A screening questionnaire was emailed to 120,000 randomly chosen Australians aged 18-30 years registered on the Australian Electoral Roll. Individuals meeting inclusion criteria were invited to a web portal where they provided consent, and undertook screening and baseline surveys. They were then interviewed via telephone to determine current GAD diagnosis using the MINI International Neuropsychiatric Interview (MINI) [29] and randomized to the trial."

Participants then completed the intervention to which they were randomised, all web programs and self-accessed.

4b-i) Report if outcomes were (self-)assessed through online questionnaires

Outcomes were self-assessed through online questionnaires at baseline, six and 12 months, with the exception of MINI caseness, which was assessed at six months.

4b-ii) Report how institutional affiliations are displayed

5) CONSORT: Describe the interventions for each group with sufficient details to allow replication, including how and when they were actually administered

5-i) Mention names, credential, affiliations of the developers, sponsors, and owners

Active Website: 10-week structured version of the Anxiety and Worry modules of the e-couch program (ecouch.anu.edu.au).

e-couch was developed by the e-hub Web Services team at the ANU Centre for Mental Health Research, and its ongoing delivery is overseen by the e-hub Management Group: Professor Kathy Griffiths (director), Ms Kylie Bennett (Collaborative Research & Development Manager), Mr Anthony Bennett (e-hub IT Manager), and Ms Julia Reynolds (e-hub Clinical Services Manager).

Control website: "an adapted version of the HealthWatch control condition developed for the ANU WellBeing study" (project team: Ms Kylie Bennett, Professor Helen Christensen, Professor Kathy Griffiths"

5-ii) Describe the history/development process

e-couch is a new program and research evidence relating to its efficacy is not yet available. However, studies testing the effectiveness of the program are currently under way.

5-iii) Revisions and updating

No changes. e-couch available at www.ecouch.anu.edu.au.

5-iv) Quality assurance methods

5-v) Ensure replicability by publishing the source code, and/or providing screenshots/screen-capture video, and/or providing flowcharts of the algorithms used

5-vi) Digital preservation

www.ecouch.anu.edu.au.

5-vii) Access

Participants accessed the active website in their own time. They did not have to pay. Access to e-couch is open, with registration.

5-viii) Mode of delivery, features/functionalities/components of the intervention and comparator, and the theoretical framework

The E-couch program is comprised of four sections - psycho-education, CBT, relaxation and exercise. Research indicates that all four components are effective in reducing anxiety levels [34,38].

Psycho-education will be covered in weeks 1 and 2. It contains information about the definition of worry; its distinction from stress and fear; the differentiation of GAD from Panic Disorder, Specific Phobia, Separation Anxiety Disorder, Adjustment Disorder, and PTSD; prevalence rates; the problem of comorbidity and information on medical, psychological, and lifestyle treatments for anxiety. The psycho-education section is modelled on mental health literacy interventions that have been shown to improve attitudes to and reduce symptoms of depression and anxiety [27]. It is based on clinical practice guidelines [39] as well as on reviews of evidence of alternative and lifestyle treatments [34]. The Cognitive Behaviour Therapy (CBT) toolkits will be introduced in weeks 3, 4, 5, 6, and 7. The CBT toolkits are designed to address typical anxious thoughts and targets worry-related thoughts and beliefs [40]. The CBT component for anxiety is based on previously developed materials which have established efficacy for anxiety cognitions and beliefs in at-risk individuals [13,41]. The third section of the E-couch intervention provides two Relaxation Exercises. These will be downloadable from the site during weeks 8 and 9 of the intervention, although they are freely available at any time. Mindful Meditation is a type of meditation which involves using awareness of breathing to keep a focus on the present moment. The Progressive Muscle Relaxation (PMR) component, aims to induce a relaxation response through systematic relaxation of the body. It involves participants progressively tensing and relaxing each muscle in their body, whilst also paying close attention to feelings of tension and relaxation. The Physical Activity intervention introduced in week 10 but lasting for longer than a week, uses walking, tailored to stages of change in participants' level of fitness.

5-ix) Describe use parameters

The Active website intervention was a 10-week structured version of the Anxiety and Worry modules of the e–couch program .

5-x) Clarify the level of human involvement

No human involvement in the e-intervention, however, certain groups also had weekly telephone reminders by the trial administrators.

5-xi) Report any prompts/reminders used

Weekly telephone reminders for 2 groups, and weekly email reminders for one group.
Emails were automated and contained messages encouraging adherence. The content of both the phone calls and the emails were similar.

5-xii) Describe any co-interventions (incl. training/support)

Attention Control Condition: "HealthWatch"
HealthWatch is an online program first developed for the ANU WellBeing Study. In the form employed in the current study it provides information about various health topics each week for 10 weeks. These cover environmental health, nutrition myths, heart health, activity, medication, the effects of temperature, oral health, blood pressure and cholesterol, calcium, and back pain. To encourage interaction, participants are also asked to respond to a number of questions about potential risk factors for anxiety. Preliminary evidence from the WellBeing research trial suggests that the site is not associated with a reduction in depressive or anxiety symptoms over time.

6a) CONSORT: Completely defined pre-specified primary and secondary outcome measures, including how and when they were assessed

"Outcome measures The primary outcome was the GAD-7[30]. Secondary outcomes were GAD caseness based on the MINI; worry, measured by the PSWQ [32]; anxiety sensitivity, as measured by ASI [33]; depression symptoms, measured by the CES-D [34]; and disability measured by Days out of Role from the US National Comorbidity Study [35]. Other measures not analysed in this paper focused on comorbidities (harmful/hazardous alcohol use as measured by AUDIT [36] or duplicate measures of depression caseness estimated by the PHQ-9 [37]), and other behaviours such as help seeking and perceived need for treatment. Outcomes were assessed at baseline, six and 12 months, with the exception of MINI caseness, which was assessed at six months."

6a-i) Online questionnaires: describe if they were validated for online use and apply CHERRIES items to describe how the questionnaires were designed/deployed

No.

6a-ii) Describe whether and how "use" (including intensity of use/dosage) was defined/measured/monitored

"Adherence to the intervention differed significantly according to condition. Participants in the reminder conditions completed the majority of the 10 modules (Active/email: 5.5 modules, Active/phone: 7.3, Control/phone: 8.3) while those who did not receive reminders completed a little over one-third (3.7 modules for both active and control; $F_{4, 477.5} = 38.1$, $p < .001$))... accounting for adherence by adjusting for module completion (i.e., testing the efficacy of the intervention) did not change these outcomes."

6a-iii) Describe whether, how, and when qualitative feedback from participants was obtained

Through the weekly telephone calls conducted by trial administrators.

6b) CONSORT: Any changes to trial outcomes after the trial commenced, with reasons

No changes.

7a) CONSORT: How sample size was determined

7a-i) Describe whether and how expected attrition was taken into account when calculating the sample size

"Sample size. We aimed, conservatively, to find an effect of 0.3 between each Active website group and the Control, based on effect sizes of 0.6 found for previous treatment and indicated prevention trials (0.6) [38, 39]. This assumes a pre-post correlation of 0.7 between scores. With 600 participants, we would have 80% power to detect effects, allowing for 15% attrition."

7b) CONSORT: When applicable, explanation of any interim analyses and stopping guidelines

Not applicable.

8a) CONSORT: Method used to generate the random allocation sequence

"Randomisation The algorithm for randomisation consisted of a stratified block design with eight strata (2 x 2 x 2) corresponding to gender, past GAD diagnosis, severity of GAD symptoms with a block size of 10. Allocation was administered via software architecture, and participants were informed of condition allocation after baseline interview."

8b) CONSORT: Type of randomisation; details of any restriction (such as blocking and block size)

"Randomisation The algorithm for randomisation consisted of a stratified block design with eight strata (2 x 2 x 2) corresponding to gender, past GAD diagnosis, severity of GAD symptoms with a block size of 10. Allocation was administered via software architecture, and participants were informed of condition allocation after baseline interview."

9) CONSORT: Mechanism used to implement the random allocation sequence (such as sequentially numbered containers), describing any steps taken to conceal the sequence until interventions were assigned

"Allocation was administered via software architecture, and participants were informed of condition allocation after baseline interview."

10) CONSORT: Who generated the random allocation sequence, who enrolled participants, and who assigned participants to interventions

As required by ICH Guideline E9, randomisation of participants to treatment groups was carried out under trial biostatisticians who were not involved in the day to day conduct of the trial.
Allocation was administered within the existing software architecture developed
by the investigators. Participants were informed that they were assigned to a condition after completing the baseline interview, and could begin the first module
one week later.

11a) CONSORT: Blinding - If done, who was blinded after assignment to interventions (for example, participants, care providers, those assessing outcomes) and how

11a-i) Specify who was blinded, and who wasn't

Participants and assessors were not blinded. Only the senior trial biostatistician was blinded to the treatment groups being considered. Furthermore, no trial biostatisticians were involved in the allocation of individuals to inventions,
administration of treatment, measuring outcomes, entering data, or assessing eligibility of participants.

11a-ii) Discuss e.g., whether participants knew which intervention was the "intervention of interest" and which one was the "comparator"

Participants were not informed which was the "intervention of interest" and which was the "comparator".

11b) CONSORT: If relevant, description of the similarity of interventions

Active and control websites were similar in length but not content.

12a) CONSORT: Statistical methods used to compare groups for primary and secondary outcomes

Primary analyses were undertaken on an intent-to-treat (ITT) basis, including all participants randomised regardless of treatment actually received or withdrawal from the trial. Mixed-model repeated measures (MMRM) analyses will be used because of the ability of this approach to include participants with missing data without using discredited techniques such as last observation carried forward. For non-inferiority components, appropriate analyses will be undertaken. These will generally not be ITT based, as this model is often anti-conservative in these circumstances.

Non-linear mixed models will be used to analyse categorical outcomes including increased caseness status and whether the participant has met the benchmark decrease of 20% from baseline at each of the follow-up assessments on the GAD-7. If necessary, multiple imputation including demographic and other background variables as predictors will be used to allow inclusion of data from all participants and not simply those with data which would permits inclusion in mixed models. Additional analyses will explore participant characteristics which moderate outcome and, if appropriate, levels of presenting severity associated with significant improvement.

12a-i) Imputation techniques to deal with attrition / missing values

"Mixed model repeated measures (MMRM) were used to accommodate missing data. Non-linear mixed models were used to analyse caseness."

12b) CONSORT: Methods for additional analyses, such as subgroup analyses and adjusted analyses

No additional analyses.

RESULTS

13a) CONSORT: For each group, the numbers of participants who were randomly assigned, received intended treatment, and were analysed for the primary outcome

"558 people were randomised to a trial condition, of whom 360 (65%) completed post-test, 303 (54%) completed 6-month follow-up and 264 (47%) completed the 12-month follow-up. Figure 1 shows the flow of participants. Sample characteristics are presented in Table 1."

13b) CONSORT: For each group, losses and exclusions after randomisation, together with reasons

CONSORT flow diagram provided.

13b-i) Attrition diagram

"Attrition was higher than expected at about 35% at post-test."

14a) CONSORT: Dates defining the periods of recruitment and follow-up

"The study protocol [28] describes trial details.".

14a-i) Indicate if critical "secular events" fell into the study period

No critical secular events.

14b) CONSORT: Why the trial ended or was stopped (early)

It was not stopped early.

15) CONSORT: A table showing baseline demographic and clinical characteristics for each group

Table provided in manuscript.

15-i) Report demographics associated with digital divide issues

Provided in Table 1. in the manuscript.

16a) CONSORT: For each group, number of participants (denominator) included in each analysis and whether the analysis was by original assigned groups

16-i) Report multiple "denominators" and provide definitions

1) Active (n=111)
2) Active / email (n=113)
3) Active / phone (n=110)
4) Control (n=111)
5) Control / phone (n=113)

16-ii) Primary analysis should be intent-to-treat

17a) CONSORT: For each primary and secondary outcome, results for each group, and the estimated effect size and its precision (such as 95% confidence interval)

"There were no differences on any of the baseline measures with the exception of ‘preference for active condition’ and employment status. Across all conditions, the most preferred program was the Active website with email reminders (38%), followed by the Control (31%), with few participants stating a preference for phone reminders (6% and 12% for Active and Control conditions, respectively). Lower preference for the Active website was found among those in the Control and Active with email conditions. Higher rates of full-time work were found among those receiving the Active website with Phone reminders and lower rates among those receiving the Control website with Phone reminders.
Adherence to the intervention differed significantly according to condition. Participants in the reminder conditions completed the majority of the 10 modules (Active/email: 5.5 modules, Active/phone: 7.3, Control/phone: 8.3) while those who did not receive reminders completed a little over one-third (3.7 modules for both active and control; F4, 477.5 = 38.1, P < .001)).
GAD-7 symptoms reduced at post-test, 6-month and 12-month follow-up periods. There were no significant differences between Group 4 (Control) and Groups 1 (Active website), 3 (Active website with email) or 5 (Control with phone) at any follow-up, although Group 3 approached significance at post-test (P =.07). However, Group 2 (Active website with phone) was associated with fewer symptoms at post-test and at 12 months than Group 4 (Control). Outcomes were unchanged after adjusting for employment status and preferences for condition. Likewise, accounting for adherence by adjusting for module completion (i.e., testing the efficacy of the intervention) did not change these outcomes. Figure 2 shows estimated marginal means of GAD-7 scores from the mixed model shown in Table 2.
There were no differences on any of the baseline measures with the exception of ‘preference for active condition’ and employment status. Across all conditions, the most preferred program was the Active website with email reminders (38%), followed by the Control (31%), with few participants stating a preference for phone reminders (6% and 12% for Active and Control conditions, respectively). Lower preference for the Active website was found among those in the Control and Active with email conditions. Higher rates of full-time work were found among those receiving the Active website with Phone reminders and lower rates among those receiving the Control website with Phone reminders.
Adherence to the intervention differed significantly according to condition. Participants in the reminder conditions completed the majority of the 10 modules (Active/email: 5.5 modules, Active/phone: 7.3, Control/phone: 8.3) while those who did not receive reminders completed a little over one-third (3.7 modules for both active and control; F4, 477.5 = 38.1, P < .001)).
GAD-7 symptoms reduced at post-test, 6-month and 12-month follow-up periods. There were no significant differences between Group 4 (Control) and Groups 1 (Active website), 3 (Active website with email) or 5 (Control with phone) at any follow-up, although Group 3 approached significance at post-test (P =.07). However, Group 2 (Active website with phone) was associated with fewer symptoms at post-test and at 12 months than Group 4 (Control). Outcomes were unchanged after adjusting for employment status and preferences for condition. Likewise, accounting for adherence by adjusting for module completion (i.e., testing the efficacy of the intervention) did not change these outcomes. Figure 2 shows estimated marginal means of GAD-7 scores from the mixed model shown in Table 2.
Data on secondary data analyses are displayed for continuous variables in Table 3. Based on the MINI assessment at six months, 16 cases of GAD were identified, comprising 6.7% from the Active groups (1, 2, and 3) and 4.5% from the Control groups (4, 5). There was no significant difference in the number of cases across these collapsed groups.
The MMRM analyses for secondary outcomes were as follows. As for the primary outcome, there were no significant overall interactions between condition and time for CES-D, PSWQ or Days out of Role. However, there was a significant interaction between condition and time for the ASI. Furthermore, there were significant effects of specific conditions at specific time points. CES-D, ASI and PSWQ scores were significantly lower for the active website with email reminders at post-test, relative to the control website condition (t389.2 = -2.5, P = .015; t368.7 = -3.4, P < .001; t371.9 = -2.4, P = .017 respectively). The decrease in ASI scores for the active/email condition remained significant at six months (t343.1 = -2.3, P = .021). In addition, Days out of Role due to anxiety was significantly decreased at 12 months (but not at post-test or six months) for the active/email condition (t398.3 = -2.4, P = .016). There was also reduced worry in the active website with phone reminders at post-test and six months, relative to the control condition (t368.6 = -2.0, P = .047; t340.2 = -2.1, P = .035 respectively)."

17a-i) Presentation of process outcomes such as metrics of use and intensity of use

"Adherence to the intervention differed significantly according to condition. Participants in the reminder conditions completed the majority of the 10 modules (Active/email: 5.5 modules, Active/phone: 7.3, Control/phone: 8.3) while those who did not receive reminders completed a little over one-third (3.7 modules for both active and control; F4, 477.5 = 38.1, P < .001))."

"Attrition was higher than expected at about 35% at post-test. Consistent with previous e health trials, data completion was higher in the control condition and was lowest for the active website condition with automated emails, a finding consistent with earlier trials."

17b) CONSORT: For binary outcomes, presentation of both absolute and relative effect sizes is recommended

"Across the three follow-up periods the Cohen’s d of the Active Website with phone ranged from .48 to -.09, the Active Website with email ranged from .41 to .09, the phone to be .19 to -.01 and the website alone to range between .00 and .19."

18) CONSORT: Results of any other analyses performed, including subgroup analyses and adjusted analyses, distinguishing pre-specified from exploratory

None performed.

18-i) Subgroup analysis of comparing only users

19) CONSORT: All important harms or unintended effects in each group

None.

19-i) Include privacy breaches, technical problems

None.

19-ii) Include qualitative feedback from participants or observations from staff/researchers

Not available.

DISCUSSION

20) CONSORT: Trial limitations, addressing sources of potential bias, imprecision, multiplicity of analyses

20-i) Typical limitations in ehealth trials

"There are a number of limitations to the present study. There was differential drop-out in conditions relative to the Control. However, no significant difference in attrition was observed between the Active website with phone reminders and Control, the comparison that was found to be effective in the trial. The completer analyses produced comparable effects to the main ITT analysis. It is possible that the generalised anxiety stream of the e-couch intervention is not effective. However, the depression stream of the e-couch website is effective, and the structure and content of the generalised anxiety stream was produced by the same team which contributed to other effective online interventions, such as MoodGYM, BluePages, and e-couch depression [40, 41]. Furthermore, large effect sizes on the GAD e-couch intervention have been observed in individuals with a GAD diagnosis (paper in preparation). The number of individuals in the current prevention study developing a diagnosis over the 12-month period following the intervention was unexpectedly low. However, elsewhere, comparable rates of 8.6% for the intervention and 4.44% in usual care groups have been reported (see [14]). These low rates may be due to regression to the mean, and to the low threshold of anxiety for recruitment to the trial. A three year follow-up is in progress to determine long-term effects. Attrition was higher than expected at about 35% at post-test. Consistent with previous e health trials, data completion was higher in the control condition and was lowest for the active website condition with automated emails, a finding consistent with earlier trials."

21) CONSORT: Generalisability (external validity, applicability) of the trial findings

21-i) Generalizability to other populations
Directly relevant to others, as participants were 18-30 year olds in the community rated at risk for developing GAD.
21-ii) Discuss if there were elements in the RCT that would be different in a routine application setting

22) CONSORT: Interpretation consistent with results, balancing benefits and harms, and considering other relevant evidence
22-i) Restate study questions and summarize the answers suggested by the data, starting with primary outcomes and process outcomes (use)
"We found evidence that a brief active website accompanied by weekly phone calls compared to an attention-matched control condition was associated with improved anxiety outcomes at post-test and that the effect persisted at 12 months. The effects were not large but reasonable considering that the comparison condition comprised an attention-matched control condition rather than a waitlist, and that none of the participants had an anxiety disorder."
22-ii) Highlight unanswered new questions, suggest future research

Other information
23) CONSORT: Registration number and name of trial registry
TRIAL REGISTRATION: ISRCTN76298775
24) CONSORT: Where the full trial protocol can be accessed, if available
Christensen, H., Griffiths, K. M., Mackinnon, A. J., Kalia, K., Batterham, P. J., Kenardy, J., ... & Bennett, K. (2010). Protocol for a randomised controlled trial investigating the effectiveness of an online e health application for the prevention of Generalised Anxiety Disorder. BMC psychiatry, 10(1), 25.
25) CONSORT: Sources of funding and other support (such as supply of drugs), role of funders
"The trial was funded by NHMRC Project Grant 525419. Helen Christensen is supported by NHMRC Fellowship 1056964. Philip Batterham is supported by NHMRC Fellowship 1035262. Kathleen Griffiths is supported by NHMRC Fellowship 1059620."
X26-i) Comment on ethics committee approval
"This study was granted ethical approval by the Australian National University
Human Research Ethics Committee (protocol number 2008/548)."
x26-ii) Outline informed consent procedures
"Individuals meeting inclusion criteria were invited to a web portal where they provided consent, and undertook screening and baseline surveys."
X26-iii) Safety and security procedures

X27-i) State the relation of the study team towards the system being evaluated
"Conflicts of interest: Griffiths is the Director of ANU e-hub self-help services which delivers the public access version of e-couch GAD and co-authored the GAD stream of e-couch; Bennett is the development manager of e-hub services. Neither derives a personal financial benefit from e-couch. "
